# Learning new words through reading: do robust spelling–sound mappings boost learning of word forms and meanings?

**DOI:** 10.1098/rsos.210555

**Published:** 2022-12-14

**Authors:** Rachael C. Hulme, Laura R. Shapiro, J. S. H. Taylor

**Affiliations:** ^1^ Aston Institute of Health and Neurodevelopment and School of Psychology, College of Health and Life Sciences, Aston University, Birmingham, UK; ^2^ Division of Psychology and Language Sciences, University College London, London, UK

**Keywords:** reading, vocabulary learning, decoding ease, orthography, semantics

## Abstract

High-quality lexical representations depend on robust representations of written form (orthography), spoken form (phonology) and meaning (semantics), and strong bonds between them. Quality of lexical representations may be affected by amount of print exposure and the form of individual words. Words that are harder to decode (print-to-sound) may lead to fuzzy representations of the orthographic and phonological forms, potentially creating less stable foundations for semantic knowledge. These factors are difficult to disentangle in natural language research; in this registered report, we experimentally manipulated decoding ease and exposure at the item level. Adults read paragraphs describing invented meanings of pseudowords. Pseudowords appeared two or six times in a paragraph, and had easy (e.g. *bamper*) or hard (e.g. *uzide*) to decode spelling–sound mappings. Post-tests assessed word-form knowledge, orthography–semantic mappings and semantic–phonology mappings. Results showed that greater decoding ease improved learning of word forms and consequently also impacted on word meanings. Higher exposure frequency improved learning of word forms but not meanings. Exposure frequency also modulated the effect of decoding ease on word-form learning, with a stronger effect of decoding ease for fewer exposures. Disentangling effects of decoding ease from print exposure has important implications for understanding potential barriers to vocabulary learning.

## 1. Introduction

Vocabulary acquisition is a lifelong process: from infancy through to adulthood, our ability to acquire new word meanings is fundamental to all learning, with lasting consequences for academic attainment and employment [1,2]. For older children and adults, the majority of new words are learned through reading (i.e. our first encounter is of their written, rather than spoken forms [3,4]). According to the lexical quality hypothesis [5,6], the quality of lexical representations is dependent on robust representations of the written form (orthography), spoken form (phonology) and meaning (semantics), and strong bonds between them. Developing these robust and tightly bonded representations requires repeated exposure to printed words, perhaps in varied contexts [5,7]. However, a less well-researched aspect of lexical quality is the extent to which the *form* of individual words influences the robustness of lexical representations. Think of a word like ‘synecdoche’: it is hard to read because the print–sound correspondences are not obvious. This difficulty in decoding print to sound may lead to a fuzzy representation of the orthography (indicated by slow recognition of the written form) and fragile links to phonology (indicated by variable pronunciations). This may in turn further disrupt the formation of tightly bonded links with semantic representations. The present study investigated whether vocabulary knowledge acquired through reading is weaker for words that are harder to decode, in comparison with the more well-established effect of exposure on vocabulary learning.

There are large individual differences in the amount people read, which inevitably leads to individual differences in the frequency with which we are exposed to printed words. Asking people how much they read can therefore provide a proxy for their print-exposure, at the participant level, which can then be compared with participant-level measures of general vocabulary knowledge, to examine the extent to which print exposure and vocabulary size are linked. For example, a large meta-analysis demonstrated that children and adolescents who choose to read more in their own time have larger vocabularies [8]. However, children who read more are likely to also be more able readers, making it difficult to determine whether it is print exposure, reading ability or both that drives vocabulary learning [9–13]. A recent behaviour–genetics study sought to answer this question [12]. Using direction of causality modelling with a large sample of twin data they showed that reading ability is highly heritable and predicts print exposure rather than vice versa [12]. While this study shows that print exposure is largely driven by reading ability, correlational designs cannot directly test the causal link between print exposure and vocabulary. Instead, they provide an indication of how individual differences in a person's overall level of print exposure influences their general vocabulary size. Experimental learning studies in which the number of exposures can be manipulated at the item level are needed to confirm the contribution of print exposure to vocabulary learning.

The focus of recent research has been on print exposure and vocabulary learning, and less is known about the influence of decoding ease. It has been proposed that efficient, more automatic decoding frees up mental resources, allowing proficient readers to focus on higher-order tasks, such as understanding the meaning of the text [14,15]. The primacy of print-to-sound decoding is also highlighted by theories of reading [16,17] in which the meaning of a word (semantics) can either be accessed directly from its written form (orthography) or indirectly via its pronunciation (phonology). For unfamiliar words, the direct orthography–semantics pathway is not yet established and is also difficult to learn due to the arbitrary nature of this mapping. When encountering new words, readers of alphabetic orthographies therefore rely on decoding orthography to phonology (utilizing the systematic relationship between letters and sounds) and then mapping from phonology to semantics [16–18]. This idea is also embedded in the self-teaching hypothesis [19], which describes how decoding the sound of a word from its written form enables whole-word (lexical) orthographic representations to be established and linked to meaning.

Vocabulary development has largely been studied from an individual differences perspective with developing readers, but, since print exposure and decoding ability are so highly correlated [8], it is difficult to isolate the relative contributions of each. By examining effects of repeated exposure and decoding difficulty at the item level we can directly ascertain whether a new vocabulary item is better acquired if it is seen more often and/or if it is easier to decode. This item-level conceptualization is justified, since greater overall print exposure necessarily implies greater exposure to individual word forms. Similarly, poorer readers necessarily make more decoding errors, hence our decoding ease manipulation captures the fact that pronunciations are more difficult to derive for some words (or some readers) than others. This conceptualization also aligns well with the lexical quality hypothesis [5,6]. If a word is encountered frequently during reading (e.g. *through* will be read more often than *evoke*) there are more opportunities to strengthen knowledge about its written form, sound and meaning, and the associations between these. Alternatively, if it is easy to map form and sound (e.g. *evoke* is easier to decode than *through* because the spelling–sound mappings are more regular) this provides a strong link between orthography and phonology and a foundation on which to build knowledge of its associated meaning. Being able to distinguish between frequency of exposure and the ease with which a word is decoded is vital for understanding the mechanisms by which we gain vocabulary knowledge from written text: is exposure sufficient to explain this relationship (i.e. greater exposure leads to greater semantic learning), or does the acquisition of word-form and meaning knowledge also depend on having a robust orthographic and phonological foundation (i.e. greater semantic learning if words are easier to decode), consistent with the lexical quality hypothesis? In the current study we used an experimental approach in which adults learned the meanings of new words through silent passage reading. We manipulated the number of times the new words were presented in each passage (two versus six exposures) and the ease with which they could be decoded, in terms of spelling-to-sound mapping (easy to decode, e.g. *bamper*, versus hard to decode, e.g. *uzide*).

Some studies have examined the effect of exposure frequency on vocabulary learning at the item level. The classic word frequency effect refers to the finding that words that are encountered more frequently are recognized more readily than words that are encountered less frequently (for a review see [20]). This is probably due to better quality lexical representations having been established for words that have been encountered more frequently, resulting in differences in processing [7]. A study of printed word learning in beginner readers showed that young readers (aged 7–9 years) can learn a new written word form after only a few exposures and use this orthographic knowledge to speed their semantic judgements, but that recognition and spelling improve with further practice [21]. Relatedly, experimental word learning studies with adults have shown that increasing the number of exposures to a word during silent reading improves learning of both the word form [22] and meaning [23]. This is in line with the lexical quality hypothesis: robust lexical representations are constructed through repeated exposure to printed words [5,7].

There have been few experimental manipulations of decoding ease. Word naming (reading aloud) and lexical decision are affected by variables such as spelling-sound consistency and orthographic neighbourhood size [24–26]. However, less is known about how these factors might affect semantic learning. Nevertheless, some studies have manipulated decoding ease in different ways to investigate the impact on learning of the word form. In a study with children aged 7–8 years, Share [27] disrupted spelling-sound decoding using irrelevant concurrent vocalizations during a lexical decision task and found that this negatively impacted orthographic learning. These findings were later replicated using similar methods with children of different age groups [28,29]. However, a lexical decision task with concurrent articulation is not akin to natural silent reading in which exposure to words is not limited to a brief time window and attention is not divided between reading the text and a secondary task. Wang *et al*. [30] manipulated the spelling-sound regularity of pseudowords that children learned through reading short stories. In post-tests of orthographic decision and spelling, performance was better for regular than irregular words [30]. However, to experimentally manipulate regularity of the spelling–sound mappings, they pre-exposed children to the phonology of the words before they read them in text (e.g. taught */fa:b/* then see written form *ferb*). This is an ecologically valid way to investigate orthographic learning in young children, who usually encounter words in text that are already familiar in oral language. However, the present study is concerned with how spelling-sound mapping difficulty affects orthographic, phonological and semantic learning when skilled silent readers encounter words for the first time in a printed text. Our decision to examine effects of decoding ease in skilled readers also provides better experimental control, since such readers have already established the systematic letter–sound relationships required for decoding, while younger readers are still developing these connections. Experimentally examining effects of decoding ease and exposures at the item level with skilled adult readers therefore allows us to establish causality of these effects in a way that would not be possible in individual differences research with developing readers.

### 1.1. The present study

In the present study, adults learned new written words (pseudowords) embedded in short paragraphs, each describing their meaning. We manipulated word learning in two ways to tap into factors that could influence the creation of lexical representations during vocabulary learning from reading. Note that the amount of semantic information provided about each new word was held constant across conditions: (i) exposure frequency—half the words appeared two times and half six times in a paragraph; (ii) decoding ease—half the words were easy to decode and half hard to decode. Decoding ease was manipulated using items from a pseudoword reading study by Mousikou *et al*. [31]: hard-to-decode pseudowords are those that they found received many different pronunciations across participants and had long response times (RTs), whereas easy-to-decode pseudowords were those that received only one pronunciation across participants and had short RTs (more details are provided in §2.5 Materials). In their study, higher pronunciation variability was predicted by lower spelling-sound consistency in sub-lexical orthographic components and a smaller orthographic neighbourhood [31]. This therefore aligns with the construct of unpredictability, identified by Schmalz *et al*. as being one of the components of orthographic depth [32,33].

We predicted that words would be learned better if they (a) received a greater number of exposures, and (b) were easier to decode. That is, we predicted that responses would be faster and more accurate for these items across all of our measures of word learning, compared with items that received fewer exposures and were harder to decode. Furthermore, we explored whether there was (c) an interaction between decoding ease and exposure. Increasing the number of exposures may be more beneficial for words that are hard versus easy to decode, and, vice versa, greater decoding ease may be more beneficial for words with fewer versus more exposures.

Vocabulary learning was assessed in multiple ways to examine our experimental predictions regarding the effects of exposure frequency and decoding ease on (i) word-form knowledge, (ii) mapping between orthography and semantics, and (iii) mapping between semantics and phonology.

(i) Quality of word-form knowledge was assessed using a four-alternative written form recognition task. On each trial participants saw one trained item and three untrained items (foils). One foil was a visual distractor for the trained word, e.g. *lunder* for the trained word *linder*. The other two foils were unrelated items that were visually similar to each other (e.g. *naffle* and *noffle*).
(ii) Quality of orthographic–semantic mappings was assessed with cued recall, in which participants typed the definition of a written trained word. As the semantic information provided in the passages was equivalent for all conditions, this determined whether our manipulations influenced participants' ability to recall word meanings from their written form.
(iii) Quality of semantic–phonology mappings was assessed by asking participants to say aloud the trained word in response to a written definition of the meaning. This determined whether the manipulations of orthographic learning had a knock-on effect on participants’ ability to recall phonological forms from their meanings.^1^

Finally, a reading aloud task served as a positive control, to confirm that our manipulation of pronunciation variability affected decoding ease in the expected direction, i.e. hard to decode words should have longer RTs (within/across participants) and more variable pronunciations (across participants). The research questions, hypotheses, sampling and analysis plan, and prospective interpretations are shown in the study design table (table 1).

**Table 1.** Study design table for the experiment.

question	hypothesis	sampling plan (e.g. power analysis)	analysis plan	interpretation given different outcomes
positive control: does decoding ease affect reading aloud for skilled adult readers?	positive control: words that were easier to decode would be read aloud more quickly than words that were harder to decode (Test 3: reading aloud)	*simR* power calculation of the effect of decoding ease on reading aloud with subset of data from Mousikou *et al*. [31]: *N* = 3 participants required to achieve at least 90% power (*α* = 0.05)	LME model with fixed effects for decoding ease, exposures, and the interaction on RT data for reading aloud	we expected a significant main effect of decoding ease, replicating Mousikou *et al*. [31] and confirming that the decoding ease manipulation successfully influenced participants' ability to generate phonology from orthography
1) do number of exposures and decoding ease affect word-form learning?	1) the written form would be learned better if words (a) received a greater number of exposures, and (b) were easier to decode (Test 1: written form recognition)	*simR* power calculations of the effects of (a) exposures and (b) decoding ease on written form recognition RT with data from pilot study: exposures *N* = 300 participants,^a^ and decoding *N* = 140 participants required to achieve at least 90% power (*α* = 0.05)	LME and logistic LME models with fixed effects for decoding ease, exposures and the interaction on RT data and binary accuracy data for written form recognition	a significant main effect of exposures would indicate that the written form is learned better with more exposures, and a significant main effect of decoding ease would indicate that the written form is learned better if words are easier to decode; non-significance of these main effects would indicate a lack of support for our hypotheses; analysis of the interaction is exploratory
2) do number of exposures and decoding ease affect learning of the orthography–semantic mappings?	2) meanings would be recalled better if words (a) received a greater number of exposures, and (b) were easier to decode (Test 2: cued recall of meanings)	*simR* power calculation of the effect of exposures on cued recall of meanings with subset of data from Hulme *et al*. [23]: *N* = 120 participants required to achieve at least 90% power (*α* = 0.05)^b^	logistic LME model with fixed effects for decoding ease, exposures, and the interaction on binary accuracy data for cued recall of meanings	a significant main effect of exposures would indicate that meanings are recalled better with more exposures, and a significant main effect of decoding ease would indicate that meanings are recalled better if words are easier to decode; non-significance of these main effects would indicate a lack of support for our hypotheses; analysis of the interaction is exploratory
3) do number of exposures and decoding ease affect learning of the semantic–phonology mappings?	3) spoken words would be recalled better if words (a) received a greater number of exposures, and (b) were easier to decode (Test 4: cued recall of words aloud)	refer to power calculation for Test 2: cued recall of meanings, which is a similar task	LME model with fixed effects for decoding ease, exposures and the interaction on Levenshtein distance score for cued recall of words aloud	a significant main effect of exposures would indicate that spoken words are recalled better with more exposures, and a significant main effect of decoding ease would indicate that spoken words are recalled better if words are easier to decode; non-significance of these main effects would indicate a lack of support for our hypotheses; analysis of the interaction is exploratory

^a^It was not feasible to recruit this number of participants for the present study, but note that we have adequate power to test the more novel hypothesis regarding the effect of decoding ease.

^b^We used data from a previous study that used exactly the same measure and exposure manipulation, this is preferable to a power calculation based on the underpowered pilot study despite the lack of a decoding ease manipulation in this case.

## 2. Method

### 2.1. Pilot study

We conducted a pilot study to validate our measures of word learning. Twenty-four participants took part in the pilot study; they were recruited from the same participant pool as for the main study (but these 24 participants did not take part in the main study). The method for the pilot study was exactly the same as for the main study. Results of the pilot study are in appendix A.1.

### 2.2. Preregistration, open materials and open data

The Registered Report Protocol Preregistration for this study is available at: https://osf.io/c84fx. A list of all stimuli used in the experiment are available on the Open Science Framework (OSF; https://osf.io/v45ge) along with details of the experimental protocol. Our experimental protocol and all tasks are available to preview through Gorilla Open Materials (https://gorilla.sc/openmaterials/86768), and the anonymized data and analysis scripts for the experiment are available on the OSF (https://osf.io/v45ge).

### 2.3. Power calculations

We carried out power calculations for our measures to establish the sample size required for the experiment. The power calculations were conducted with the *simr* package [34] in R (v. 4.0.0 [35]), using datasets from previous studies with similar outcome measures where available, or our pilot data where there was no suitable existing dataset. We conducted power calculations to achieve 90% power to detect a main effect of decoding ease or number of exposures (*α* = 0.05; see table 1 for a summary of the study design details including the power analyses). These calculations indicated a sample size of at least 140 participants was required (which was calculated for the written form recognition measure) and we therefore aimed to recruit 144 participants in total (to allow for 18 participants per version). Further details of the power calculations are in appendix A.2.

### 2.4. Participants

We initially recruited 144 participants; however, we had to remove seven participants' data from the written form recognition task, as their performance was not significantly above chance (see §2.9 Data exclusion and transformation). We therefore recruited an additional seven participants to ensure sufficient power as specified by our power calculations.

In total 151 adult participants participated in the study, with 18 or 19 participants per version (*M*_age_ = 30.16 years, s.d. = 6.09; 96 female, 55 male). Included participants were native English speakers aged 18–40 years with normal or corrected-to-normal vision and who had not been diagnosed with a hearing, reading or language disorder. Participants were recruited online through the Prolific website (www.prolific.co).

### 2.5. Materials

#### 2.5.1. Novel word forms

Novel word forms were items from Mousikou *et al*. [31]. Their study analysed stress assignment, pronunciation and naming latencies for a set of 915 pseudowords, read aloud by 41 adults. Pseudoword pronunciations were found to vary across participants and this was quantified using the *H*-statistic [31,36], a measure of entropy accounting for the proportion of participants that gave each alternative pronunciation. Higher pronunciation variability was predicted by lower spelling–sound consistency in sub-lexical orthographic components and a smaller orthographic neighbourhood [31]. Furthermore, naming times were slower for items with a higher *H*-statistic suggesting that these items were also difficult for individual readers to decode. A recent vocabulary learning study by Ricketts *et al*. [37] also used *H* to index the spelling–sound consistency of multi-syllabic words. We, therefore, decided to use pseudowords from Mousikou *et al*. and assume that items with a high *H*-statistic are harder to decode than those with a low *H*-statistic.

Sixteen pseudowords were selected from those used by Mousikou *et al*. [31]. Mousikou *et al*. [31] identified subsets of their pseudowords that were given only a single pronunciation (*H* = 0; *n* = 50), or many different pronunciations (mean *H* = 3.11; range of number of pronunciations: 12–22; *n* = 54) across 41 participants. We selected eight items for our easy decoding condition from the set of 50 pseudowords with only a single pronunciation (e.g. *bamper*) and eight items for our hard decoding condition from the set of 54 pseudowords with highly variable pronunciations (e.g. *uzide*). We began by selecting items with the fastest/slowest mean naming times respectively across Mousikou *et al*.'s [31] participants, and removed and replaced any items that were too similar to an already-selected item (items differing by a Levenshtein distance of less than 3). Additionally, we selected the same number of prefixed/suffixed items in the set of words for the easy and hard decoding conditions. The characteristics of the final set of 16 pseudowords are shown in table 2 (see table 3 in appendix A.3 and table 4 in appendix A.4 for pronunciations of the pseudowords generated by participants in our pilot study and main study respectively).

**Table 2.** Descriptive statistics for the easy and hard target words taken from Mousikou *et al*. [31].

	easy words (e.g. *bamper*)	hard words (e.g. *uzide*)
number of letters	6.13 (0.35)	6.63 (0.92)
orthographic neighbours	2.75 (3.15)	0.13 (0.35)
spelling-to-sound consistency (*H*; averaged across syllables)^a^	0.37 (0.34)	0.46 (0.35)
number of pronunciations	1.00 (0.00)	14.38 (2.00)
*H* (pronunciation variability)	0.00 (0.00)	3.07 (0.32)
reading aloud RTs (ms)	675.50 (73.42)	1015.88 (102.62)

^a^The spelling-to-sound consistency measure given by Mousikou *et al*. [31] was expressed using the *H*-statistic, so that higher values indicate less consistency in the spelling-to-sound mapping.

For each of the pseudoword stimuli, a visual foil was derived for use in the written form recognition task. These were created by changing a single letter from the target word (e.g. *balper* for *bamper*). While previous studies using a similar task have also included a homophonic (phonological) foil (e.g. [30]), in our study word learning is through silent reading without prior exposure to the words' phonology, so a visual distractor alone is more appropriate. To ensure that our foil items were plausible word forms in accordance with English spelling rules and that they were not considered to be less plausible word forms than the target pseudowords we carried out a short pretest. A separate group of 23 monolingual native British English speakers (*M*_age_ = 36.70 years, s.d. = 13.30; 20 female, 3 male) rated how likely it would be for each target pseudoword or foil to be a new word in English on a scale from 1 (*highly unlikely*) to 7 (*highly likely*). The results of a paired-samples *t*-test showed that there was no difference in perceived wordlikeness between the target pseudowords (*M*_rating_ = 3.98, s.d. = 1.27) and the foil items (*M*_rating_ = 4.08, s.d. = 1.16; *p* = 0.585). This was the case for both the easy pseudowords (target items: *M*_rating_ = 4.96; foil items: *M*_rating_ = 4.91) and the hard pseudowords (target items: *M*_rating_ = 2.99; foil items: *M*_rating_ = 3.24).

#### 2.5.2. Novel word meanings

The 16 novel word meanings used in the present study were selected from Rodd *et al*.'s [38] paragraphs describing new word meanings. These new word meanings comprised hypothetical innovations, natural or social phenomena, invented objects, and technical and colloquial terms. The paragraphs were matched for length (*M_length_* = 91.69 words, s.d. = 4.53) and contained about five pieces of information about the new meanings. Rodd *et al*.'s [38] paragraphs were adapted so that each word would appear with its new meaning six times in the high-exposure condition, and two times in the low-exposure condition. For the low-exposure condition, the paragraphs were altered so that all instances of a word apart from the first and final exposures in the paragraph were replaced with pronouns, synonyms, or simply omitted. This allowed us to keep the amount of semantic information to be learned the same between the two exposure conditions. An example paragraph is shown below for two of the conditions: the easy, low-exposure condition (*invill* with two exposures) and the hard, high-exposure condition (*uzide* with six exposures). Note that participants only saw one of these paragraphs, with the other versions used to balance which word meaning was presented in each condition across participants. (The target words and/or the pronouns/synonyms replacing them were not highlighted in any way in the paragraphs that participants read in the study.) All of the stimulus materials are available through the OSF (https://osf.io/v45ge).

No recording device is smaller than the **invill**. The **device** is virtually undetectable and while it can be hidden, **it** may even go unnoticed in plain sight. Each **one** contains a tiny camera that is remote activated and that sends a video feed back to the controller. Ingeniously, **the units** are mobile and can be moved around by remote control when they are required to get a better view. However, with the technology comes a high price, which currently limits the use of **invills** to that of government intelligence services.

No recording device is smaller than the **uzide**. The **uzide** is virtually undetectable and while it can be hidden, the **uzide** may even go unnoticed in plain sight. Each **uzide** contains a tiny camera that is remote activated and that sends a video feed back to the controller. Ingeniously, the **uzide** units are mobile and can be moved around by remote control when they are required to get a better view. However, with the technology comes a high price, which currently limits the use of **uzides** to that of government intelligence services.

### 2.6. Design

The independent variables of decoding ease (easy versus hard) and number of exposures (two versus six) were manipulated within participants. Eight versions of the experiment were created to counterbalance the factors of decoding ease, number of exposures and specific item meaning across participants. Each participant was trained on half the total number of stimuli (eight items), as our pilot study and previous research [38,39] suggested this to be a reasonable number of new meanings to expect participants to learn in a single session. Each novel word meaning was paired with a hard word form in half of the versions and with an easy word form for the other half, and each word-form–meaning pairing appeared in the low-exposure condition in half of the versions, and the high-exposure condition for the other half. We randomly and evenly assigned participants to one of the eight versions of the experiment: 18 or 19 participants were assigned to each version. The dependent variables were accuracy and RT in the written form recognition task, RT in reading aloud, and accuracy in cued recall of meanings and recalling words aloud from their meanings (scored as Levenshtein distance).

### 2.7. Procedure

**Figure 1.**
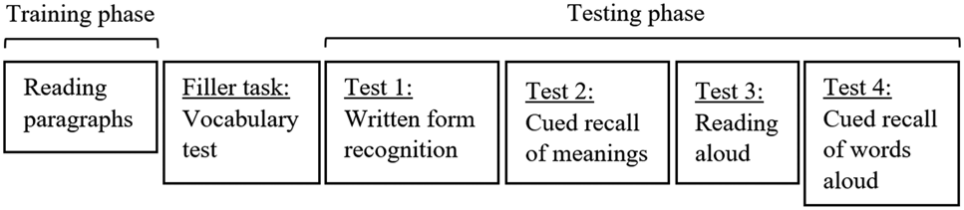
Overview of the order of the tasks in the experiment. The test tasks were administered in the same order for all participants. The set order of the test measures was decided through careful consideration of the potential impact of each task on the subsequent tasks. For example, the written form recognition task comes first because the cued recall of meanings and reading aloud measures provide additional exposures to the orthographic form.

The experiment was conducted using Gorilla experimental software (www.gorilla.sc [40]). A schematic of the order of tasks included in the experiment can be found in figure 1. At the start of the experiment participants completed a practice run-through of all tasks with three practice items. This followed exactly the same procedure as the main experiment now described, except that the filler task was omitted. For the training phase participants were instructed to carefully read a series of paragraphs describing the fictitious meanings of made-up words, and to try to learn them. Participants were informed that their memory for these new words and their meanings would be subsequently tested. The paragraphs describing the new words and their meanings were displayed on-screen one at a time. Participants clicked to move on to the next paragraph and were not able to go back to reread paragraphs. The paragraphs describing the new word meanings appeared in a randomized order for all participants.

After reading the paragraphs, participants completed a brief filler task between the training and testing phases. This was to counteract possible recency effects, i.e. better memory for new words encountered towards the end of the training phase. The filler task was the Shipley vocabulary test [41]. For each of the 40 items in the vocabulary test, participants were required to choose one word that had the same meaning as a target word from four options.

#### 2.7.1. Measures of word learning

##### 2.7.1.1. Test 1: written form recognition

A written form recognition task assessed participants' knowledge of the orthographic form of the trained target words. On each trial, participants saw a target item (e.g. *bamper*), a related foil item (e.g. *balper*), and two unrelated foil items (e.g. *invill* and *invilt*) which were items that were not trained for that participant. Since related foils differed by one letter and also in pronunciation, we note that decisions could be based on purely orthographic knowledge or may be supported by the phonology participants derived during learning. The aim of this task was not to distinguish between these two possibilities but to assess learning of word-form, rather than semantic, knowledge. The inclusion of the unrelated foils allowed us to exclude participants with at-chance performance due to poor learning resulting in guessing. Target and foil items appeared on-screen in a randomized order (left to right) and participants were asked to select the word they learned by pressing the corresponding key (d, f, j or k). They were instructed that the words they did not learn may look similar to the words they did learn. Trial order was randomized for each participant. RT and accuracy were recorded.

##### 2.7.1.2. Test 2: cued recall of meanings

Cued recall assessed participants' ability to recall the word meanings from their written form. On each trial, participants saw the written form of a trained item and typed its meaning. They were encouraged to provide as much detail as possible and to guess if they were unsure. If they were not able to remember anything for a given word, they were instructed to write ‘don't know’. Item order was randomized for each participant.

##### 2.7.1.3. Test 3: reading aloud

A reading aloud task assessed the mapping between orthographic and phonological forms of the new words. Participants were presented with the written form of each new word, and asked to read it aloud as quickly and clearly as possible. Responses were recorded using a computer microphone (participants were encouraged to use a head-mounted microphone where available). The order the words were presented was randomized across participants.

The reading aloud task served as a positive control. Data from the mega-study of disyllabic pseudoword reading by Mousikou *et al*. [31] showed that pseudowords that had many different pronunciations across participants (our hard items) were read aloud more slowly than pseudowords that had only a single pronunciation (our easy items). This was a large effect with a mean difference of around 200 ms in naming RT between the hard and easy words. We expected to replicate this pattern of results in our reading aloud task.

##### 2.7.1.4. Test 4: cued recall of words aloud

The fourth test was cued recall of the words' spoken form from written definitions. This task assessed mappings between semantic and phonological knowledge of the new words. Participants read single-sentence definitions of each of the trained items and were asked to say aloud the corresponding trained word as clearly and accurately as possible. The order of presentation of items was randomized across participants.

### 2.8. Data coding

Data from the written form recognition task were coded as 1 when the target word was correctly selected, or 0 when one of the foils was selected. Responses from the cued recall of meanings task were manually coded for accuracy by the experimenter as either 1 (correctly recalled) or 0 (incorrectly recalled). The responses were leniently coded as correct if at least one of the semantic features of a new word meaning was correctly recalled, this was deemed to be the best approach in previous research using this task [23,39].

Reading aloud RTs were derived by hand-marking the acoustic onsets of the audio-recorded responses using CheckVocal [42]. Additionally, recordings were phonetically transcribed into the speech assessment methods phonetic alphabet (SAMPA)^2^ to record the different pronunciations participants gave for the words. Audio-recorded responses for the cued recall of words aloud task were transcribed in the same way. Responses for this task were coded for accuracy by taking the Levenshtein distance between the SAMPA-transcribed response and the SAMPA-transcribed response the participant gave for the same item in the reading aloud task. We used the Levenshtein distance measure to score the spoken responses from the cued recall of words aloud task rather than a simple accuracy measure (see [37] for a similar approach to scoring responses on an orthographic test). This is because there is no ‘correct’ or ‘incorrect’ pronunciation for these novel pseudowords, as participants had to derive the phonology from the orthography themselves during silent reading. Furthermore, as the hard-to-decode words were designed to be more variable in pronunciation, we were concerned that a simple binary accuracy measure would be biased against the items in this condition.

### 2.9. Data exclusion and transformation

Forty-five participants were excluded and replaced during recruitment due to having incomplete data for technical reasons, and seven participants were excluded and replaced as they admitted writing down answers in the question about cheating at the end of the study. Excluded participants were replaced by new participants to achieve the total number of participants required for the study. With respect to data removal from individual tasks, we removed seven participants' data from the written form recognition task as they were not significantly above chance (3/8 correct), no participants were removed from the cued recall of words aloud and reading aloud tasks for problems with audio recordings, and data from one participant were removed from the cued recall of words aloud task due to misunderstanding the instructions (they read aloud the definitions instead of recalling the words). We visually inspected the distributions of data from our tasks that recorded RTs and only excluded extreme outlier trials from our dataset (12 outliers were removed from the written form recognition task and four outliers were removed from the reading aloud and cued recall of words aloud tasks). Analysis of the RT data for the written form recognition task was of correct trials only. The assumptions of homoscedasticity and normality are violated in the raw RT data for the written form recognition and reading aloud measures, so we log- and inverse-transformed (invRT = 1000/rawRT) the data and compared these with the raw RT data. Histograms showing the distributions of these data and scatterplots of the residuals versus fitted values were used to compare the raw, log- and inverse-transformed data; the log-transformed RTs most closely met the assumptions of homoscedasticity and normality and were used for the analyses.

### 2.10. Analysis procedure

The accuracy data for responses on written form recognition and cued recall of meanings were analysed using logistic linear mixed effects (LME) models using the *lme4* package [44] and R statistical software (v. 4.0.0 [35]). The RT data for written form recognition and reading aloud, and the Levenshtein distance data for cued recall of words aloud were analysed using LME models using the same software and package. The contrasts for the factors of interest were defined using deviation coding for decoding ease (hard: −0.5 versus easy: 0.5) and number of exposures (two: −0.5 versus six: 0.5), with the interaction between decoding ease and number of exposures coded by multiplying the contrasts for these two factors.

Following recommendations by Barr *et al*. [45], the first attempt to fit a model^3^ for each of our measures used the maximal random effects structure. The models contained fixed effects for decoding ease, number of exposures and the interaction. They also included random effects of participants (with slopes for the random effects of decoding ease, number of exposures and the interaction by participants) and random effects of items (with a slope for the random effect of number of exposures by word forms). For all measures the maximal model did not converge without singularity (overfitting), so we took the following steps in turn until a final model (and all of the necessary nested models with individual fixed effects removed) converged without overfitting. First, we removed the correlations between the random slopes and the intercepts; second, we removed the random intercepts leaving in the slopes. None of the models for any of our measures converged without overfitting after these steps, so we proceeded to the third step of following a data-driven forward best-path model selection procedure starting with the simplest model with only random intercepts and incrementally adding in each of the random slopes one at a time. To do this we built a model containing each random slope individually and compared each of these models (that converged without overfitting) with the simple random-intercepts-only model using likelihood ratio (LHR) tests to see if any of these models gave a significantly improved fit (using *α* of 0.2 [45,46]). If any of the models with an individual random slope was a significant improvement to the model with only random intercepts, the random slope from the model whose LHR test obtained the smallest *p*-value was included first. This model was subsequently compared with models containing this random slope plus any other random slope individually that improved the random-intercepts-only model, and so on until an added slope did not significantly improve model fit. Where none of these models was a significant improvement on the random-intercepts-only model, then we used that model as the final model for the analysis. The random effects were not allowed to correlate highly with one another; this was taken as a sign that the model was overfitted and not appropriate for the data, and in this case the next best model that converged without overfitting (with the most complex random effects structure possible) was used instead. The final models used to analyse each measure are specified in the Results section. We determined significance of the fixed effects and interaction using LHR tests comparing the full final model with models with each of the fixed effects/interaction of interest removed in turn (but leaving in an interaction involving a factor of interest that had been removed) and leaving the random effects structure intact.

#### 2.10.1. Positive control

The LME model for RT in the reading aloud task assessed the effect of decoding ease, number of exposures, and their interaction on pseudoword naming. We expected to see a significant main effect of decoding ease, replicating Mousikou *et al*. [31] and confirming that the decoding ease manipulation successfully influenced participants' ability to generate phonology from orthography.

#### 2.10.2. Hypothesis testing

The research questions, hypotheses, sampling and analysis plan, and prospective interpretations are shown in the study design table (table 1). The LME models described above were used to test the hypotheses that (i) words would be learned better with more exposures (a significant main effect of number of exposures), (ii) words that were easier to read would be learned better (a significant main effect of decoding ease), and (iii) to explore whether there was a significant interaction between decoding ease and number of exposures. If the interaction was significant in any of the models, then we conducted follow-up analyses to determine the nature of the interaction. These follow-up tests allowed us to assess whether the number of exposures affects hard-to-decode words more than easy-to-decode words, and/or whether decoding ease benefits words encountered less as compared with more frequently.

## 3. Results

### 3.1. Reading aloud (positive control)

**Figure 2.**
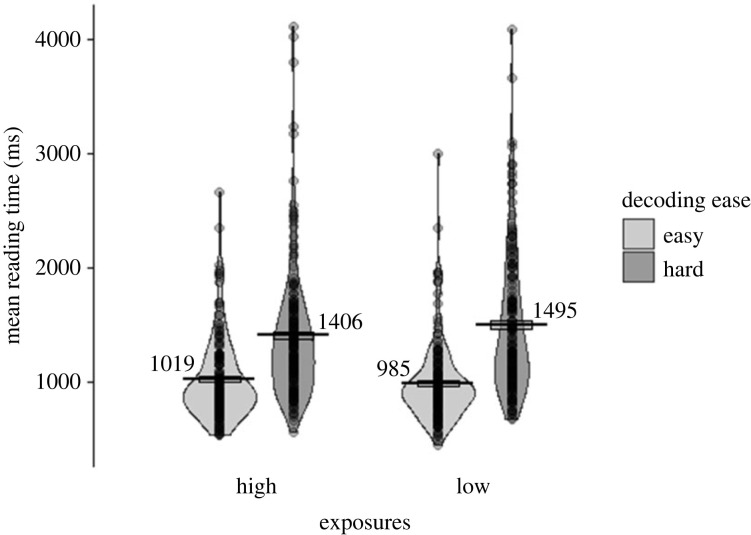
RT (in milliseconds for speech onset) in reading words aloud (Test 3). Data are displayed by number of exposures (high, low; x-axis), and decoding ease (easy in light grey, hard in dark grey). Points represent participants' condition means, each line shows the mean across participants for that condition, the boxes around the mean show the standard error (corrected for the within-participants design), and the violin shows the density.

RTs (speech onset) for the reading aloud task are shown in figure 2. A model with the following structure was fitted to the log-transformed speech onset times: *lmer(logRT ∼ 1* *+* *DecodingEase* *+* *Exposures* *+* *Interaction* *+* *(1|Participant)* *+* *(1|Item)).*^4^ As predicted, easy words were read aloud more quickly than hard words [$\chi^{2}(1)=29.42$, *p* < 0.001], thus replicating the finding of Mousikou *et al*. [31] and confirming that the decoding ease manipulation successfully influenced participants' ability to generate phonology from orthography. We did not make any prediction regarding effects of the number of exposures or the interaction on reading aloud times. There was no significant effect of exposures [$\chi^{2}(1)=0.25$, *p* = 0.617], although there was a significant interaction between exposures and decoding ease [$\chi^{2}(1)=4.32$, *p* = 0.038]. However, exploratory follow-up simple effects analyses showed that easy words were read aloud more quickly than hard words for both a high [$\chi^{2}(1)=19.55$, *p* < 0.001; *α* = 0.025] and low [$\chi^{2}(1)=28.25$, *p* < 0.001; *α* = 0.025] number of exposures.

### 3.2. Written form recognition

**Figure 3.**
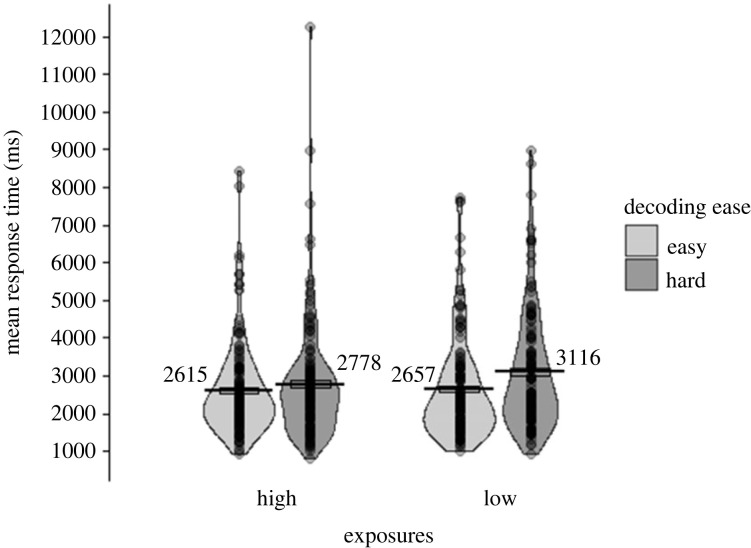
RT (in milliseconds) in written form recognition (Test 1) for correct responses only. Data are displayed by number of exposures (high, low; x-axis), and decoding ease (easy in light grey, hard in dark grey). Points represent participants’ condition means, each line shows the mean across participants for that condition, the boxes around the mean show the standard error (corrected for the within-participants design), and the violin shows the density.

The RT data for the written form recognition task (figure 3) were log-transformed and fitted with the following model: *lmer(logRT ∼ 1* *+* *DecodingEase* *+* *Exposures* *+* *Interaction* *+* *(1|Participant)* *+* *(1|Item))*. As predicted, words that were easy to decode were recognized faster than hard words [$\chi^{2}(1)=4.55$, *p* = 0.033], and words with more exposures were recognized faster than those with fewer exposures [$\chi^{2}(1)=4.00$, *p* = 0.046]. There was also a significant interaction between exposures and decoding ease [$\chi^{2}(1)=11.58$, *p* = 0.003]. Exploratory follow-up simple effects analyses showed that easy words were recognized more quickly than hard words when there was a low number of exposures [$\chi^{2}(1)=8.59$, *p* = 0.003; *α* = 0.025], but there was no effect of decoding ease for a high number of exposures [$\chi^{2}(1)=0.42$, *p* = 0.519; *α* = 0.025].

**Figure 4.**
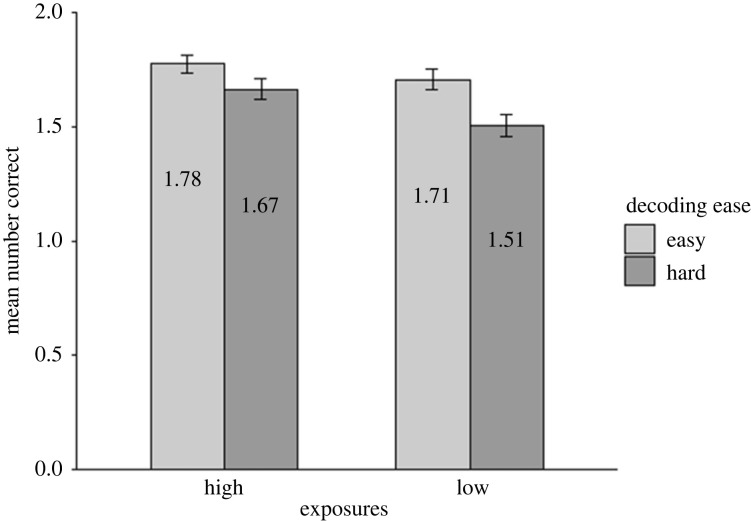
Mean number of correct responses across participants in written form recognition (Test 1). Data are displayed by number of exposures (high, low; x-axis), and decoding ease (easy in light grey, hard in dark grey). Error bars show the standard error (corrected for the within-participants design).

The accuracy data for the written form recognition task (figure 4) were analysed using the following model: *glmer(Accuracy ∼ 1* *+* *DecodingEase* *+* *Exposures* *+* *Interaction* *+* *(1|Participant)* *+* *(1|Item))*. As predicted, and consistent with the RT data, words that were easier to decode were recognized more accurately than hard words [$\chi^{2}(1)=4.76$, *p* = 0.029], and words with more exposures were recognized more accurately than those with fewer exposures [$\chi^{2}(1)=4.10$, *p* = 0.043]. Although the accuracy data showed a similar trend as for the RT data, there was no significant interaction between number of exposures and decoding ease [$\chi^{2}(1)=0.41$, *p* = 0.524].

### 3.3. Cued recall of meanings

**Figure 5.**
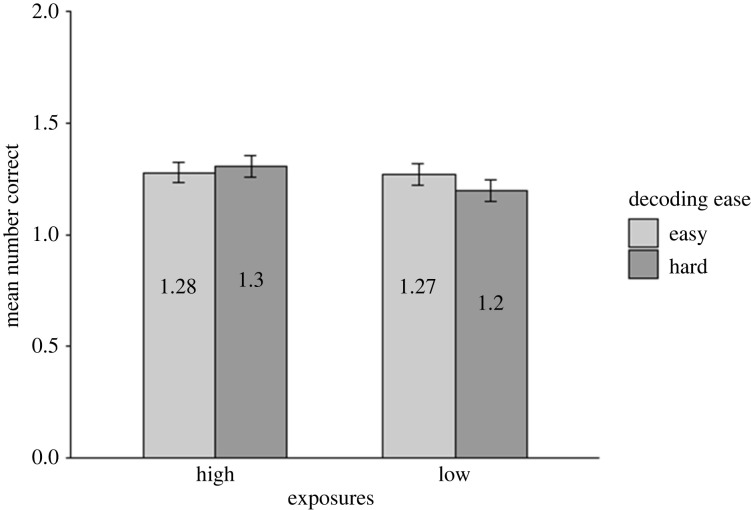
Mean number of correct responses across participants in cued recall of meanings (Test 2). Data are displayed by number of exposures (high, low; x-axis), and decoding ease (easy in light grey, hard in dark grey). Error bars show the standard error (corrected for the within-participants design).

The accuracy data for the cued recall of meanings task (figure 5) were fitted with the following model for the analysis: *glmer(Accuracy ∼ 1* *+* *DecodingEase* *+* *Exposures* *+* *Interaction* *+* *(1* *+* *Interaction|Participant)* *+* *(1|Item))*. Contrary to our predictions, there was no significant main effect of decoding ease [$\chi^{2}(1)=0.14$, *p* = 0.713] nor number of exposures [$\chi^{2}(1)=1.73$, *p* = 0.189] on accuracy in cued recall of meanings. There was also no significant interaction between number of exposures and decoding ease [$\chi^{2}(1)=0.13$, *p* = 0.723].

### 3.4. Cued recall of words aloud

**Figure 6.**
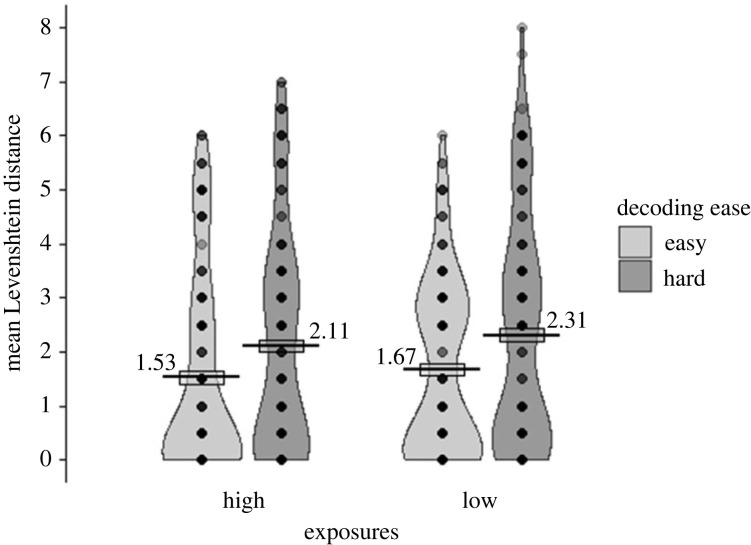
Levenshtein distance comparing the SAMPA-transcribed responses of participants in cued recall of words aloud (Test 4) with their response for the same item when reading it aloud (Test 3), a Levenshtein distance of 0 indicates exactly the same pronunciation. Data are displayed by number of exposures (high, low; x-axis), and decoding ease (easy in light grey, hard in dark grey). Points represent participants' condition means, each line shows the mean across participants for that condition, the boxes around the mean show the standard error (corrected for the within-participants design), and the violin shows the density.

The Levenshtein distance data for the cued recall of words aloud task (figure 6) were analysed using the following model: *lmer(LevDist ∼ 1* *+* *DecodingEase* *+* *Exposures* *+* *Interaction* *+* *(1|Participant)* *+* *(1|Item))*. As predicted, words that were easier to decode were recalled aloud from their meanings more accurately (had a lower Levenshtein distance from reading aloud response) than hard words [$\chi^{2}(1)=7.47$, *p* = 0.006]. However, contrary to our predictions there was no significant main effect of number of exposures [$\chi^{2}(1)=2.43$, *p* = 0.119]. There was also no significant interaction between number of exposures and decoding ease [$\chi^{2}(1)=0.04$, *p* = 0.845].

## 4. Discussion

In this study, we investigated how learning word forms and meanings is influenced by two key factors: the ease with which written words can be decoded into sounds, and the amount of exposure to the printed form. Although there is good evidence that increased exposure enhances the quality of lexical representations, there has been little research into how decoding ease influences the formation of lexical representations. We experimentally manipulated these two factors and our results showed that greater ease of decoding improved learning of word forms. Decoding ease also had an influence on word-meaning learning, with greater decoding ease facilitating stronger mappings between semantics and phonology. Furthermore, the effect of decoding ease on word-form learning was modulated by the number of exposures, with a stronger effect for fewer exposures. Greater exposure frequency also independently improved learning of word forms, but not word meanings.

The results of our written form recognition test indicate that making it easier to decode print to sound facilitates orthographic learning. As we predicted, participants were faster and more accurate at recognizing the written form of words that were easy to decode than words that were hard to decode. The effect of decoding ease on word-form learning was also modulated by the number of exposures. Easy words were recognized faster than hard words when there was a low number of exposures, but there was no effect of decoding ease for a high number of exposures. The accuracy data showed a similar trend, although the interaction was not significant, suggesting that accuracy was a less sensitive measure than response times, despite a fair amount of interindividual variability. Relatedly, although our experiment was powered to detect an effect of decoding ease on response time for written form recognition, it is possible that this task lacked sensitivity to detect more subtle differences in written form recognition accuracy.

The significant effect of decoding ease on written form recognition speed and accuracy is consistent with previous studies with children in which decoding was experimentally disrupted through irrelevant concurrent vocalizations [27–29], or by manipulating the spelling–sound regularity of trained pseudowords [30]. The validity of our decoding ease manipulation was confirmed by the results of the reading aloud (positive control) task, in which easy words were read aloud faster than hard words, as we predicted and replicating findings of Mousikou *et al*. [31]. This demonstrates that our decoding manipulation was effective at disrupting orthography–phonology mappings. To our knowledge, this is the first study to experimentally disrupt decoding during silent reading for proficient adult readers, in order to examine the impact on learning of word forms and meanings.

There was also evidence that decoding ease affected acquired knowledge of word meanings. When cued with a definition of the word meaning, participants recalled aloud the easy words more accurately than the hard words. This is in absence of any exposure to the orthographic forms of the words in this task. One explanation for this could be that stronger orthography–phonology mappings for the easy-to-decode words led to more robust phonological representations that were then more readily accessible to participants when responding to the semantic cues [16,17]. This finding is consistent with the lexical quality hypothesis [5,6], and suggests that the acquisition of word-form and meaning knowledge depends on having a robust orthographic and phonological foundation.

However, there was no evidence that decoding ease had any influence on the mappings between orthography and semantics. When participants were cued with the written word form, there was no significant effect of decoding ease on recall of word meanings. The lack of an effect of decoding ease on cued recall of word meanings is difficult to interpret. The data showed a trend toward slightly lower accuracy for hard words than easy words in the low-exposure condition but not for a high number of exposures, although neither the main effect of decoding ease nor the interaction was significant. It is possible that the effect of decoding ease was too subtle to disrupt orthography–semantics mappings in this task. However, another possibility is that this task lacked sensitivity to detect such an effect. Recent work by Ricketts *et al*. [37] has highlighted the value of using more graded measures to assess word knowledge. We coded responses for our cued recall of meanings task using a binary measure of accuracy, so it is possible that partial knowledge of word meanings may be obscured in our measure. Future work could use a more sensitive graded measure, such as the number of details recalled about a meaning, to try to capture depth of word-meaning knowledge.

As we predicted, the number of exposures to a word also independently affected word-form knowledge. Participants were faster and more accurate at recognizing the written forms of words with a high number of exposures than those with a low number of exposures. This finding is consistent with previous work that has shown that increasing the number of exposures to a word during silent reading improves learning of its form [22].

By contrast to the effect of exposures on word-form knowledge, and contrary to our predictions, there was no evidence of an effect of exposures on word-meaning knowledge in either the cued recall of meanings or cued recall of words aloud tasks. The lack of an effect of exposures on orthography–semantics mappings is surprising, as it goes against Hulme *et al*. [23] who found better word-meaning knowledge for words with a greater number of exposures during story reading. Hulme *et al*. [23] observed a significant linear, incremental increase in meaning recall accuracy across two, four, six, and eight exposures, so we would expect a similarly strong difference between our high- and low-exposure conditions. However, in the study by Hulme *et al*. [23] participants encountered words in the context of longer, naturalistic stories that spanned several pages of text, in contrast to the brief paragraphs that participants read in the present study. As such, exposures to the words in our paragraphs were massed closer together than in longer stories. Previous research on semantic priming has demonstrated that massed and spaced exposures to words differentially affect word-meaning priming [47]: spaced repetitions provide a boost to priming that massed exposures do not. It is therefore possible that our high- and low-exposure conditions behaved more similarly to each other than in the study by Hulme *et al*. [23] due to the more massed presentation in shorter passages. A further effect of massing versus spacing may also have been present *within* the paragraphs in the present experiment. In the high-exposure condition, the six exposures to a word occurred in consecutive sentences, or even twice within the same sentence. By contrast, in the low-exposure condition, the two occurrences of a word were more spaced, occurring in the first and last sentence of a paragraph. As temporal spacing is known to benefit semantic learning [48], this may have cancelled out the disadvantage of the lower number of exposures, leading to the null findings for word-meaning learning. Relatedly, Betts *et al*. [47] note that it is possible that synonyms replacing the target words may cause participants to reactivate the target words themselves. This possibility is also more likely in our study than in that of Hulme *et al*. [23] as the target words were visible on the screen at the same time as the synonyms. These factors may have diminished the impact of our exposures manipulation on semantic learning. It is, however, unclear why spacing effects may have affected semantic but not word-form learning, as written form recognition was faster and more accurate for words with a high number of exposures.

Our findings concerning effects of decoding ease and exposure on word learning through reading at the item level have important implications for vocabulary learning throughout the lifespan. Our participants were skilled adult readers, yet still showed effects of decoding ease on both orthographic form learning and recalling the phonological form from its meaning. This effect of decoding is likely to be especially relevant for students learning technical terms. For words with unusual grapheme–phoneme correspondences such as ‘heuristic’ or ‘seismology’, many exposures would be needed before word-form knowledge is robust enough such that these terms can be included in a written answer or used in a spoken response. This is consistent with the lexical quality hypothesis and highlights the importance of strong orthographic–phonological connections for developing high-quality meaningful representations. One might assume that these connections are already secure in skilled adults, yet even adults will be confronted with words that are challenging to decode, and our data shows that this will hinder the development of robust lexical representations.

Extrapolating to the person level, our findings suggest that both difficulty with decoding print to sound and the amount of print exposure an individual gets could affect their acquisition of new word-form knowledge. Those who have difficulties with decoding may struggle not only with reading words but also with remembering their spellings and recalling the correct pronunciation for a concept in oral language. This may be especially the case for words to which they have had less exposure in print.

### 4.1. Summary and conclusion

The present study indicates that greater ease of decoding print to sound facilitates word-form learning, even for skilled adult readers. The effect of decoding ease on word-form learning was also modulated by the number of exposures, so more exposures may be necessary for acquiring hard-to-decode words. This can have knock-on effects for word-meaning learning, since we showed that phonological forms were recalled less accurately from their meanings for hard-to-decode words. We interpret these findings in the context of the lexical quality hypothesis [5,6]: successful acquisition of word-form and meaning knowledge depends on having a foundation of robust orthographic and phonological representations. However, our study is unable to rule out a second possible mechanistic account regarding processing constraints. Greater ease of decoding may facilitate word learning because fewer cognitive resources are required to decode easy words, allowing more attention to be focused on acquiring the word meaning. Disentangling these possibilities is difficult behaviourally; future research examining the time course of reading and word learning (e.g. eye movements or EEG) could perhaps help to tease them apart. Regardless of the precise reason for the present findings, they have clear implications for language learning. An accurate representation of a word's form is essential for correct use of the term. For example, in the context of science vocabulary, there are many terms with distinct meanings that have similar forms (e.g. ‘reflection’ versus ‘refraction’), such that their meaning is inextricably linked to the accuracy of the word-form representation. Therefore, educators should be aware of how difficult words are to decode when introducing new vocabulary to students, and give extra exposures when this is likely to be a barrier, even if they are working with skilled readers.

## Figures and Tables

**Figure 7.**
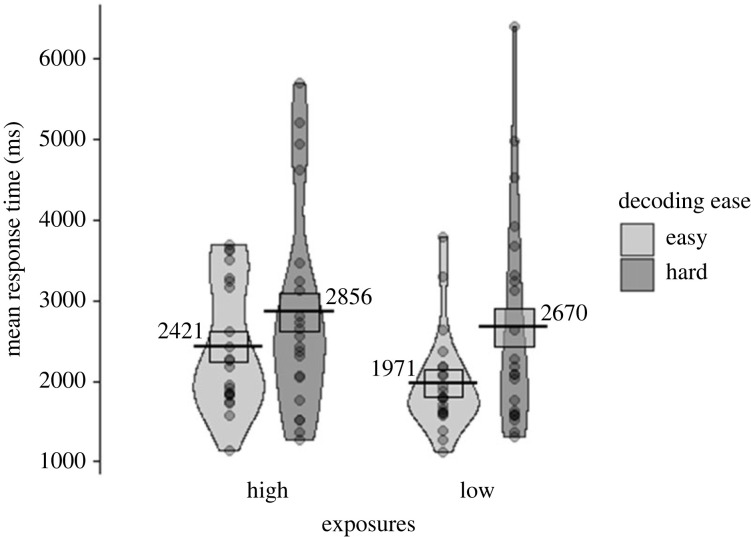
RT (in milliseconds) in written form recognition (Test 1) for correct responses only. Data are displayed by number of exposures (high, low; x-axis), and decoding ease (easy in light grey, hard in dark grey). Points represent participants’ condition means, each line shows the mean across participants for that condition, the boxes around the mean show the standard error (corrected for the within-participants design), and the violin shows the density.

**Figure 8.**
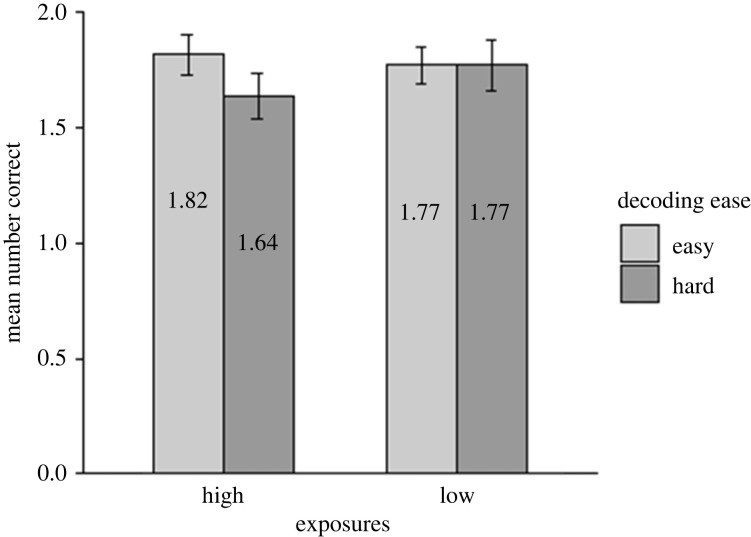
Mean number of correct responses across participants in written form recognition (Test 1). Data are displayed by number of exposures (high, low; x-axis), and decoding ease (easy in light grey, hard in dark grey). Error bars show the standard error (corrected for the within-participants design).

**Figure 9.**
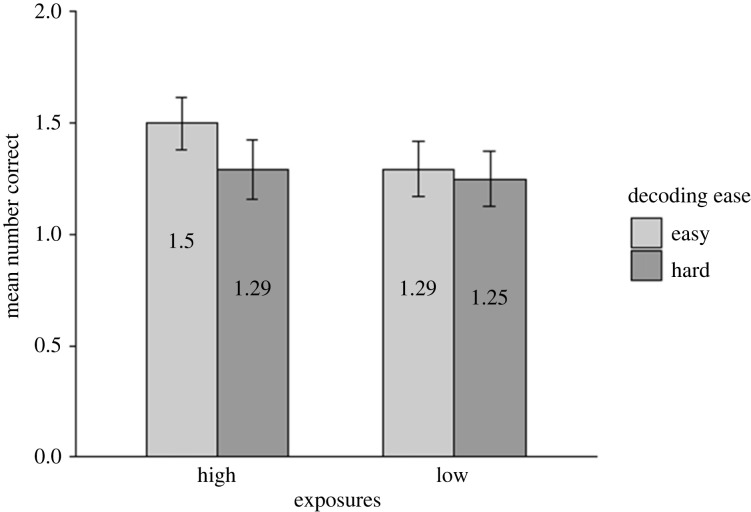
Mean number of correct responses across participants in cued recall of meanings (Test 2). Data are displayed by number of exposures (high, low; x-axis), and decoding ease (easy in light grey, hard in dark grey). Error bars show the standard error (corrected for the within-participants design).

**Figure 10.**
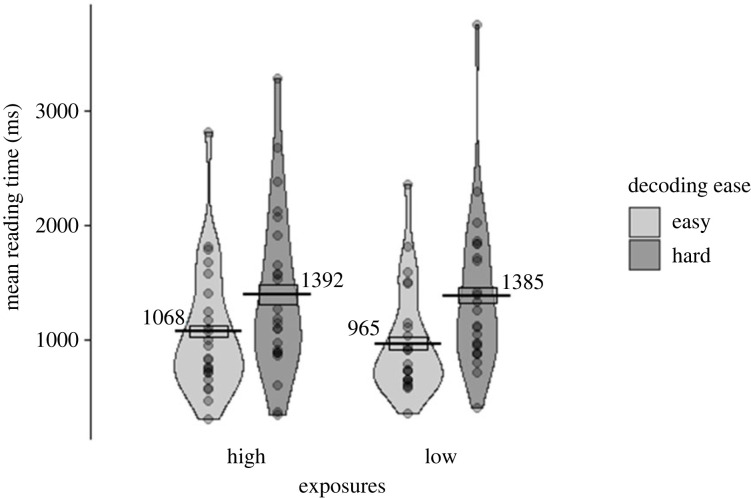
RT (in milliseconds for speech onset) in reading words aloud (Test 3). Data are displayed by number of exposures (high, low; x-axis), and decoding ease (easy in light grey, hard in dark grey). Points represent participants' condition means, each line shows the mean across participants for that condition, the boxes around the mean show the standard error (corrected for the within-participants design), and the violin shows the density.

**Figure 11.**
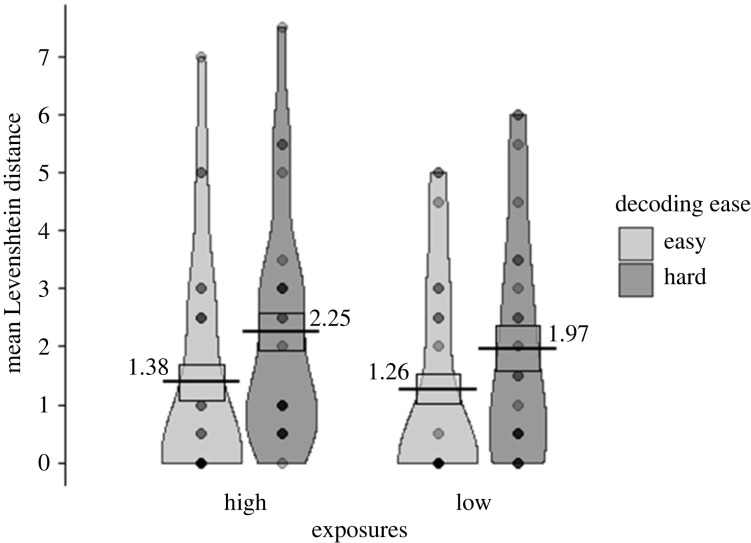
Levenshtein distance comparing the SAMPA-transcribed responses of participants in cued recall of words aloud (Test 4) with their response for the same item when reading it aloud (Test 3), a Levenshtein distance of 0 indicates exactly the same pronunciation. Data are displayed by number of exposures (high, low; x-axis), and decoding ease (easy in light grey, hard in dark grey). Points represent participants’ condition means, each line shows the mean across participants for that condition, the boxes around the mean show the standard error (corrected for the within-participants design), and the violin shows the density.

**Figure 12.**
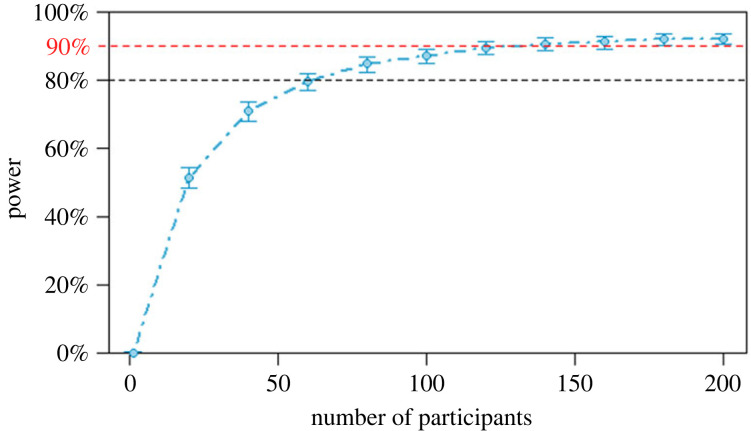
Power curve for the main effect of decoding ease on written form recognition RT (Test 1) for data from the pilot study. The level of power is displayed on the y-axis, and the number of participants required to achieve a certain level of power is displayed on the x-axis. The simulation calculated the average estimated level of power (and 95% confidence interval) for 11 sample sizes between 1 and 200, based on 1000 simulations each. We aimed to achieve 90% power (*α* = 0.05).

**Figure 13.**
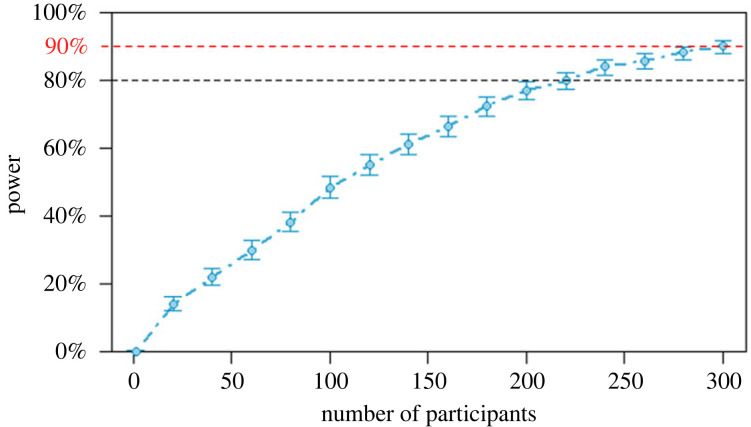
Power curve for the main effect of exposures on written form recognition RT (Test 1) for data from the pilot study. The level of power is displayed on the y-axis, and the number of participants required to achieve a certain level of power is displayed on the x-axis. The simulation calculated the average estimated level of power (and 95% confidence interval) for 16 sample sizes between 1 and 300, based on 1000 simulations each. We aimed to achieve 90% power (*α* = 0.05).

**Figure 14.**
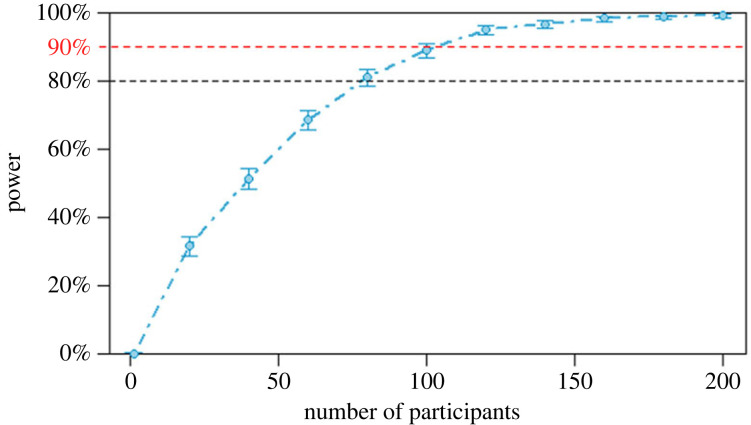
Power curve for the main effect of exposures on cued recall of meanings accuracy (Test 2) for a subset of data from Hulme *et al*. [23] comparing two and six exposures. The level of power is displayed on the y-axis, and the number of participants required to achieve a certain level of power is displayed on the x-axis. The simulation calculated the average estimated level of power (and 95% confidence interval) for 11 sample sizes between 1 and 200, based on 1000 simulations each. We aimed to achieve 90% power (*α* = 0.05).

**Figure 15.**
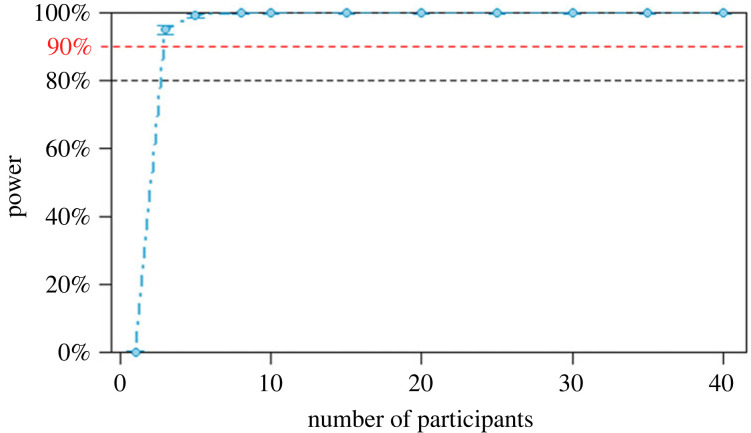
Power curve for the main effect of decoding ease on reading aloud RT (Test 3) for a subset of data from Mousikou *et al*. [31] of items that were used in the present study, with eight items per participant to match our design. The level of power is displayed on the y-axis, and the number of participants required to achieve a certain level of power is displayed on the x-axis. The simulation calculated the average estimated level of power (and 95% confidence interval) for 11 sample sizes between 1 and 40, based on 1000 simulations each. We aimed to achieve 90% power (*α* = 0.05).

## Data Availability

Our data and analysis scripts are available on the Open Science Framework (OSF) at: https://osf.io/v45ge.

## Footnotes

1 Note that it was not possible to use phonological forms as a cue for meaning recall since different participants may have different ideas about how the trained words are pronounced.

2 Transcriptions were made in a slightly modified version of SAMPA that allowed a single sound to be encoded as a single symbol, e.g. the ‘or’ */ɔ:/* sound in *tactord* was transcribed as 9 (see appendix A of [43]).

3 The BOBYQA optimizer was used for all models.

4 The random effects structure for the final model for each measure was determined by following the steps outlined in §2.10 Analysis procedure.

## Appendix A.

**A.1. Pilot study results**

Results for the pilot study (*N* = 24, with two participants excluded from Test 1) are shown in figures 7–11.^5^ Due to the small sample size, we did not run statistical tests to assess differences. The pattern of results for our positive control (reading words aloud; Test 3; figure 10) closely replicated Mousikou *et al*. [31] who also found a difference in mean RT of around 300 ms between the easy and hard-to-decode conditions. Furthermore, as expected there was less variability in pronunciation of our easy items across participants (mode = 1) than our hard items (mode = 3, 4 and 5), see table 3 in appendix A.3 for all pronunciations of the stimulus words from the pilot study. Pilot data are available on the OSF (https://osf.io/v45ge).

**A.2. Power calculations**

Power calculations for analyses using mixed effects models are not straightforward due to difficulties in obtaining standardized effect sizes (see [49]). We used the *simr* package [34] in R (v. 4.0.0 [35]) to calculate the sample size we needed to achieve 90% power based on Monte Carlo simulations, which requires existing datasets. The scripts and data files used for these calculations are available on the OSF (https://osf.io/v45ge). LME models were fitted using the *lme4* package [44] in R; models for all analyses used a simplified, intercepts-only random effects structure with random effects of participants and items to allow models to converge without singularity (overfitting).

It is recommended not to base power calculations on effect size estimates obtained from small underpowered pilot studies [50,51]. However, due to the novelty of our study we could not find suitable existing datasets for all of our measures, therefore the power calculation for Test 1 (written form recognition) was carried out using our pilot data (22 participants for this task), so caution in its interpretation is required. The power calculation for Test 1 indicated that we needed 140 participants to have 90% power to find a significant effect (*α* = 0.05) of decoding ease on written form recognition RT (figure 12). The power calculation for the effect of exposures on written form recognition RT indicated that we would need 300 participants (figure 13); however, it was not feasible to recruit this number of participants for the present study.

The power calculation for accuracy in Test 2 (cued recall of meanings) was carried out on a subset of data from Hulme *et al*. [23] with an exposures manipulation that was the same as that in the current study (two versus six exposures). This indicated that we needed around 120 participants to have 90% power to find a significant effect (*α* = 0.05) of exposures on cued recall (figure 14). The data from Hulme *et al*. did not enable a power calculation for the effect of decoding ease for Test 2.

For Test 3 (reading aloud), the power calculation for the effect of decoding ease was carried out on a subset of data from Mousikou *et al*. [31] with the same items as in the present study, and eight items assigned per participant to match our design. The effect of decoding ease on RT in the Mousikou dataset was very large, so the power calculation indicated that we needed only three participants to have 90% power to find a significant effect (*α* = 0.05) of decoding ease on reading aloud RT in the present study (figure 15). As this task was a positive control for the manipulation of decoding ease we did not do a power calculation for the effect of exposures.

For Test 4 (cued recall of words aloud) we are not aware of any previous studies that have used a similar task with adult participants; however, this task is similar to our Test 2 (cued recall of meanings) albeit in a different modality, so we referred to the power calculation for Test 2 for this task. Therefore, we aimed to recruit 144 participants in total for our study, which would allow for an even number of participants in each of the eight versions of the experiment (18 participants per version).

**A.3. Pronunciations of stimulus words from pilot study**

Pronunciations of the stimulus words in the reading aloud task (Test 3) from the pilot study are shown in table 3.

**Table 3.** Pronunciations of the stimulus words in Test 3 (reading aloud) of the pilot study phonetically transcribed into a slightly modified version of the speech assessment methods phonetic alphabet (SAMPA), with the frequency of each pronunciation shown (number of participants who gave that pronunciation). N.B. Frequencies for some of the stimulus words do not sum to 12 due to a small number of trials with missing data (corrupted/blank audio files).

stimulus word	decoding ease condition	pronunciations	frequency
bamper	easy	bamp@	12
habble	easy	hab@l	12
invill	easy	InvIl	11
linder	easy	lInd@	11
noffle	easy	nQf@l	11
noffle	easy	nQs@l	1
sottle	easy	sQt@l	12
tactord	easy	takt9d	11
tactord	easy	taktQd	1
wimble	easy	wImb@l	11
danest	hard	danEst	6
danest	hard	d1nIst	2
danest	hard	d@nEst	1
danest	hard	d1nEst	1
danest	hard	danIst	1
danest	hard	danIsts	1
geveld	hard	gEvEld	4
geveld	hard	_EvEld	2
geveld	hard	_IvEld	1
geveld	hard	gEvuld	1
geveld	hard	gEvVld	1
geveld	hard	gUvEld	1
geveld	hard	gVvEld	1
gingrem	hard	gINgrEm	8
gingrem	hard	_INgrEm	2
gingrem	hard	_INg3Em	1
gingrem	hard	gIngrUm	1
knisple	hard	nIsp@l	10
knisple	hard	nIps@l	1
knisple	hard	nIsp	1
perphise	hard	p3f2s	5
perphise	hard	p3fis	3
perphise	hard	p#fIs	1
perphise	hard	p3pis	1
perphise	hard	pafis	1
rudgerb	hard	rUdg3b	10
rudgerb	hard	rUd_3b	1
rudgerb	hard	rUdg3	1
sychlom	hard	s2klQm	7
sychlom	hard	S2lQm	1
sychlom	hard	sIklQm	1
sychlom	hard	SlQm	1
uzide	hard	uz2d	6
uzide	hard	juz2d	3
uzide	hard	Ins2d	1
uzide	hard	juzid	1
uzide	hard	us2d	1

**A.4. Pronunciations of stimulus words**

Pronunciations of the stimulus words in the reading aloud task (Test 3) from the main study are shown in table 4.

**Table 4.** Pronunciations of the stimulus words in Test 3 (reading aloud) of the experiment phonetically transcribed into a slightly modified version of the speech assessment methods phonetic alphabet (SAMPA), with the frequency of each pronunciation shown (number of participants who gave that pronunciation). N.B. Four outlier trials were removed, one from each of the following items: noffle, wimble, knisple and uzide.

stimulus word	decoding ease condition	pronunciations	frequency
bamper	easy	bamp@	68
bamper	easy	bamp3	6
bamper	easy	amp@	1
bamper	easy	bamb@	1
habble	easy	hab@l	72
habble	easy	1b@l	1
habble	easy	h@b@l	1
habble	easy	habul	1
invill	easy	InvIl	63
invill	easy	InvEl	4
invill	easy	invIl	3
invill	easy	Invil	2
invill	easy	Inv2l	1
invill	easy	Invul	1
invill	easy	InvUl	1
linder	easy	lInd@	61
linder	easy	lInd3	7
linder	easy	lind@	4
linder	easy	lInd@r	2
linder	easy	lind3	1
noffle	easy	nQf@l	74
noffle	easy	n#f@l	1
sottle	easy	sQt@l	74
sottle	easy	skQt@l	1
sottle	easy	sQtul	1
tactord	easy	takt9d	69
tactord	easy	takt3d	1
tactord	easy	taktk9d	1
tactord	easy	taktud	1
tactord	easy	taktUd	1
tactord	easy	tatt9d	1
tactord	easy	tEkt9d	1
wimble	easy	wImb@l	70
wimble	easy	wimb@l	3
wimble	easy	wEmb@l	1
danest	hard	danEst	53
danest	hard	d1nEst	16
danest	hard	d1ndIst	1
danest	hard	d1nIst	1
danest	hard	d9nEst	1
danest	hard	danEs	1
danest	hard	dansEt	1
danest	hard	dinEst	1
geveld	hard	gEvEld	24
geveld	hard	gUvEld	16
geveld	hard	_EvEld	9
geveld	hard	gEvUld	6
geveld	hard	gIvEld	6
geveld	hard	_EvUld	3
geveld	hard	_UvEld	3
geveld	hard	_gEvUld	1
geveld	hard	_VvEld	1
geveld	hard	g@vEld	1
geveld	hard	g1vIld	1
geveld	hard	g8vEld	1
geveld	hard	gEvElt	1
geveld	hard	gIvUld	1
geveld	hard	h@vEld	1
gingrem	hard	gINgrEm	27
gingrem	hard	gINgrUm	21
gingrem	hard	gIngrEm	7
gingrem	hard	_INgrEm	4
gingrem	hard	_IngrEm	3
gingrem	hard	gINgr3m	3
gingrem	hard	_IN_2m)	1
gingrem	hard	_IN_rEm	1
gingrem	hard	_INgrUm	1
gingrem	hard	gENgrEm	1
gingrem	hard	gErUm	1
gingrem	hard	gINgrEM	1
gingrem	hard	gIngrEn	1
gingrem	hard	gINrUm	1
gingrem	hard	guNgrEm	1
gingrem	hard	INUm	1
gingrem	hard	JIngrEm	1
knisple	hard	nIsp@l	47
knisple	hard	k@nIsp@l	6
knisple	hard	nIps@l	6
knisple	hard	kInsp@l	2
knisple	hard	nIspUl	2
knisple	hard	hInsp@l	1
knisple	hard	k@nIsm@l	1
knisple	hard	kusp@l	1
knisple	hard	lIsp@l	1
knisple	hard	n2sp@l	1
knisple	hard	nEksp@l	1
knisple	hard	nIgsp@l	1
knisple	hard	nIp@l	1
knisple	hard	nIspil	1
knisple	hard	nIspIl	1
knisple	hard	nispUl	1
knisple	hard	nIsUlp	1
perphise	hard	p3f2s	43
perphise	hard	p3fis	20
perphise	hard	p3p2s	3
perphise	hard	p2f2s	1
perphise	hard	p3fIs	1
perphise	hard	p3fis1	1
perphise	hard	p3fr2s	1
perphise	hard	p3pis	1
perphise	hard	p3sf2s	1
perphise	hard	p8pis	1
perphise	hard	prUs2s	1
perphise	hard	v3fIs	1
rudgerb	hard	rUdg3b	45
rudgerb	hard	rudg3b	13
rudgerb	hard	rU_3b	3
rudgerb	hard	rUd_3b	3
rudgerb	hard	ru_3b	1
rudgerb	hard	rUd-3b	1
rudgerb	hard	rUd3b	1
rudgerb	hard	rUdg3d	1
rudgerb	hard	rUdg3rbi	1
rudgerb	hard	rUdg8	1
rudgerb	hard	rudJ3b	1
rudgerb	hard	rug3b	1
rudgerb	hard	rUg3b	1
rudgerb	hard	rUg3bJ	1
rudgerb	hard	rug3d	1
rudgerb	hard	rUJ_3b	1
sychlom	hard	s2klQm	33
sychlom	hard	sIklQm	11
sychlom	hard	SlQm	5
sychlom	hard	s2klUm	4
sychlom	hard	sIklUm	4
sychlom	hard	s2lQm	3
sychlom	hard	s2kl5m	2
sychlom	hard	2klQm	1
sychlom	hard	S@klQm	1
sychlom	hard	s2klEm	1
sychlom	hard	S2klQm	1
sychlom	hard	s2kQlm	1
sychlom	hard	s2kQm	1
sychlom	hard	S2lQm	1
sychlom	hard	s2SlQM	1
sychlom	hard	SgQl	1
sychlom	hard	siklQm	1
sychlom	hard	sIklQM	1
sychlom	hard	sIkQlm	1
sychlom	hard	sk2lUm	1
uzide	hard	uz2d	34
uzide	hard	juz2d	30
uzide	hard	juzid	3
uzide	hard	uzId	2
uzide	hard	u2zd	1
uzide	hard	usd1	1
uzide	hard	Uz2d	1
uzide	hard	uzid1	1
uzide	hard	uzId1	1
uzide	hard	Uzid8	1
